# *Luticola tenera* sp. nov. (Diadesmidaceae, Naviculales)—A New Diatom from the Soil of the State Nature Reserve “Bastak” (Jewish Autonomous Region, Russia)

**DOI:** 10.3390/life13091937

**Published:** 2023-09-20

**Authors:** Veronika B. Bagmet, Shamil R. Abdullin, Arthur Yu. Nikulin, Vyacheslav Yu. Nikulin, Andrey A. Gontcharov

**Affiliations:** Federal Scientific Center of the East Asia Terrestrial Biodiversity, Far Eastern Branch of the Russian Academy of Sciences, 159, 100-Letia Vladivostoka Prospect, Vladivostok 690022, Russia; chara1989@yandex.ru (V.B.B.); artyrozz@mail.ru (A.Y.N.); nikulinvyacheslav@gmail.com (V.Y.N.); gontcharov@biosoil.ru (A.A.G.)

**Keywords:** diatom, new species, chloroplast *rbc*L gene, light and scanning electron microscopy, morphological characteristics, life cycle, temperate monsoon climate zone

## Abstract

Diatoms inhabit different aquatic and non-aquatic environments, including soils. The naviculoid genus *Luticola* is widespread in various habitats and accounts for 264 species that are only based on morphological and morphometric characteristics. These parameters can greatly vary during the life cycle, making the species very similar to each other and complicating their unambiguous identification. During a study on soil algal diversity in the Russian Far East (Jewish Autonomous Region), we isolated a strain of naviculoid diatom and examined it using an integrative approach (phylogenetic, morphological, ultrastructural data, and life cycle). Phylogenetic analyses, based on chloroplast *rbc*L gene data, showed affinity of the new strain with the genus *Luticola*. Our alga shares morphological features typical of the genus members but differs from them by having valves with a larger width and hook-shaped external proximal raphe ends deflected to the side opposite the stigma. It was revealed that the strain reproduces via two types of sexual reproduction—isogamy and cis-anisogamy. Based on these phenotypic traits, we described the new isolate as *Luticola tenera* sp. nov.

## 1. Introduction

Diatoms are the largest phylum of algae and account for over 18,500 species identified to date [https://www.algaebase.org; accessed on 19 July 2023], [1]. They inhabit different aquatic and non-aquatic ecosystems, including soils, playing an important role in ecosystems and for humans. Representatives of the Bacillariophyta are promising objects for industrial cultivation because they synthesize fatty acids, polysaccharides, pigments, nanosized siliceous structures, phenolic compounds, etc. [2,3,4,5,6].

The genus *Luticola* D.G. Mann was established in 1990 by D.G. Mann in Round et al. [7] to accommodate *Navicula* s.l. species characterized by the following combination of morphological features: uniseriate striae with more-or-less round poroids on the valve face; the presence of a single stigma; filiform raphe with deflected, bent, or hooked central and terminal endings, as well as a longitudinal canal positioned between the valve wall.

The genus includes widespread soil, aerophytic, freshwater, and brackish water species. In 2013, Levkov et al. [8] revised the genus *Luticola* and described 91 taxa new to science. In total, their monograph contained descriptions of about 200 species. Since then, a number of new species have been attributed to the genus (e.g., [9,10,11,12,13,14,15,16,17]). To date, according to the Algaebase (https://www.algaebase.org; accessed on 11 August 2023), the genus *Luticola* includes 264 species that are widely distributed throughout these regions of the world: Eurasia [18,19,20], Europe [8], South America [11,21], islands in the southern Indian Ocean [9], Africa [17], Madagascar [12], Asia [10,22], and Antarctica [23,24,25].

Despite the constantly growing number of the new taxa, there is little molecular genetic data on the members of *Luticola*. The GenBank database (https://www.ncbi.nlm.nih.gov; accessed on 11 August 2023) contains 19 sequences representing 5 species, 2 forms, and 4 species only identified based on the genus: *Luticola muticopsis* f. *evoluta* West and G.S. West, *L. muticopsis* f. *capitata* G.W.F. Carlson, *L. ventricosa* (Kütz.) D.G. Mann, *L. sparsipunctata* Levkov, Metzeltin and A. Pavlov, *L. permuticopsis* Kopalová and VandeVijver, *L. goeppertiana* (Bleisch) D.G. Mann ex Rarick, S. Wu, S.S. Lee, and Edlund. All members of this genus were based on the morphological and morphometric characters, while some features of the valve ultrastructure, in particular the cingulum, were often ignored.

It is often noted that species delimitation based on morphology alone can lead to errors in taxonomic identification. This issue is mainly due to the polymorphic nature of some morphological characters. The size and shape of the valves change during the life cycle of diatoms, and teratological forms appear [7,26,27,28,29,30]. Therefore, it is important to record the cardinal points and any changes in the morphological and morphometric characteristics of the taxon during ontogeny, which cannot be achieved without observing the sexual process. This aspect has not been practically studied in the genus *Luticola*. So far, the sexual process has only been examined in *L. dismutica* (Hustedt) D.G. Mann [31] and *L. poulickovae* Levkov, Metzeltin and A. Pavlov [29].

During a study of soil algal diversity in the temperate monsoon climate zone in the Russian Far East (State Nature Reserve “Bastak”, Jewish Autonomous Region), a strain of naviculoid diatom was isolated and examined using an integrative approach, including the study of phylogenetic, morphological, and ultrastructural data and analysis of life cycle. It led us to differentiate this strain from other members of the genus *Luticola* and describe it as a new species, namely *Luticola tenera*.

## 2. Materials and Methods

### 2.1. Sampling and Culture Conditions

A sample of waterlogged soil was collected in the State Nature Reserve “Bastak” (Jewish Autonomous Region, Russia; 48°25′59.5″ N 134°13′18.1″ E) via standard methods [32]. A clone of naviculoid diatom was isolated via the micro-pipette method [33] and incubated into 40 mm Petri dishes with liquid nutrient medium Dm [34] under the following conditions: 20–22 °C, photon fluence 17.9–21.4 μmol photons∙m^−2^ s^−1^, and 16:8 h light:dark cycle. The clone was kept in the culture collection of the Laboratory of Botany in the Federal Scientific Center of East Asian Terrestrial Biodiversity, Russian Federation (clone number VCA-254), and its dried biomass was deposited in the Herbarium of the Federal Scientific Center of East Asian Terrestrial Biodiversity, Russia (exsiccatum numbers VLA-CA-1377).

### 2.2. Microscopy

The morphology and morphometrics of the cells were studied using an Olympus BX53 light microscope (LM) (Olympus Corporation, Tokyo, Japan) equipped with Nomarski DIC optics and an Olympus DP27 digital camera (Olympus Corporation, Tokyo, Japan), as well as a Merlin scanning electron microscope (SEM) (Carl Zeiss, Jena, Germany). Frustules were cleaned via oxidation with hydrogen peroxide, rinsed several times with distilled water, and mounted in a Pleurax medium. The material was dried onto brass stubs and coated with a gold–palladium (Au–Pd, 6:4) alloy for SEM. The morphometric data were analyzed using the software package Statistica 10.0 and Microsoft Office Excel 2007.

Chloroplast fluorescence in living cells was examined using LSM 510 META and LSM 710 LIVE confocal laser scanning microscopes (CLSM, Carl Zeiss, Jena, Germany) at the Instrumental Centre of Biotechnology and Gene Engineering of FSCEATB FEB RAS. Cells were stained with DAPI (Molecular Probes, Eugene, OR, USA) to visualize the position of the nucleus [35]. Files with the 3D-captured images were recorded and analyzed via LSM 510 Release v.4.2 and ZEN 2011 software.

### 2.3. Mating Experiments

Sexual reproduction in our clone was observed during cultivation under the conditions described above. Mixed cells were examined daily with an inverted light microscope CK30-F200 (Olympus Corporation, Tokyo, Japan) for three weeks in February–March 2022. Living cells, auxosporulation, and the stages of sexual reproduction were observed and described using LM following the methods described by Poulíčková and Mann [36] and Poulíčková et al. [35].

### 2.4. DNA Extraction, Amplification, and Sequencing

For DNA analysis, the culture was harvested during the exponential growth phase and concentrated via centrifugation. Total genomic DNA was extracted as described previously by Abdullin et al. [37]. For the amplification of the plastid encoded *rbc*L gene, the following primers were used: DPrbcL1 (5′-AAGGAGGAADHHATGTCT-3′) and DPrbcL7 (5′-AAASHDCCTTGTGTWAGTYTC-3′; [38]). PCR was performed using an Encyclo Plus PCR kit (Evrogen, Moscow, Russia) with a T100 Thermal Cycler (Bio-Rad Laboratories, Inc., Hercules, CA, USA). The PCR products were purified using the ExoSAP-IT PCR Product Cleanup Reagent (Affymetrix Inc., Santa Clara, CA, USA) and sequenced in both directions using an ABI 3500 genetic analyzer (Applied Biosystems, Waltham, MA, USA) with a BigDye terminator v.3.1 sequencing kit (Applied Biosystems, Waltham, MA, USA), and the same primers were used for PCR. Sequences were assembled via the Staden Package v.1.4 [39]. The contig sequence covering the partial *rbc*L gene was deposited in GenBank under accession number OR326853.

### 2.5. Alignment and Dataset

In order to clarify the phylogenetic position of the new strain, a dataset including 74 sequences of the Naviculales Bessey was used. Three centric diatom species were chosen as the outgroup. The dataset was enriched using accessions showing similarity to the sequence obtained from our strain, as inferred from the BLAST searches (https://blast.ncbi.nlm.nih.gov/Blast.cgi; accessed on 20 July 2023). The sequences were aligned in the SeaView program [40].

### 2.6. Phylogenetic Analysis

Maximum likelihood (ML) analysis was carried out using PAUP 4.0b10 [41]. Bayesian inference (BI) was performed using MrBayes 3.1.2 [42]. The GTR+I+G nucleotide substitution model was selected as the optimal for both methods according to the Akaike Information Criterion (AIC; [43]) in jModelTest [44]. ML analysis was carried out using heuristic searches via a branch-swapping algorithm (tree bisection and reconnection). For BI, Markov chain Monte Carlo (MCMC) analysis was run for 2,000,000 generations. Trees were sampled every 100 generations to yield 20,000 trees, including 5000 trees (25%) that were discarded in the burn-in phase. Tree convergence and stationary was accessed in the Bayesian analysis by plotting the likelihood values in Tracer v1.7.1 [45]. The robustness of the trees was estimated via bootstrap percentages (BP; [46] and posterior probabilities (PP) in BI. BP < 50% and PP < 0.95 were not considered. The ML-based bootstrap analysis was performed using RAxML v.7.7.1 web service (http://embnet.vital-it.ch/raxml-bb/; accessed on 2 February 2023; [47]).

## 3. Results

We examined a strain of naviculoid diatom isolated from the soil sample (Jewish Autonomous Region, Russia) using both phenotypic features and *rbc*L sequence data. Our strain possessed a combination of phenotypic traits typical of the genus *Luticola*: distinctly punctate striae; a conspicuously isolated pore in the central area, commonly referred to as a ‘stigma’; and internal, longitudinal, and marginal canals on each side of the valve. Phylogenetic analyses, based on chloroplast *rbc*L gene data, also showed the affinity of the new strain with the genus *Luticola*. Hereafter, we refer to this strain as a new species: *Luticola tenera*.

### 3.1. Taxonomic Analysis

**Figure 1:**
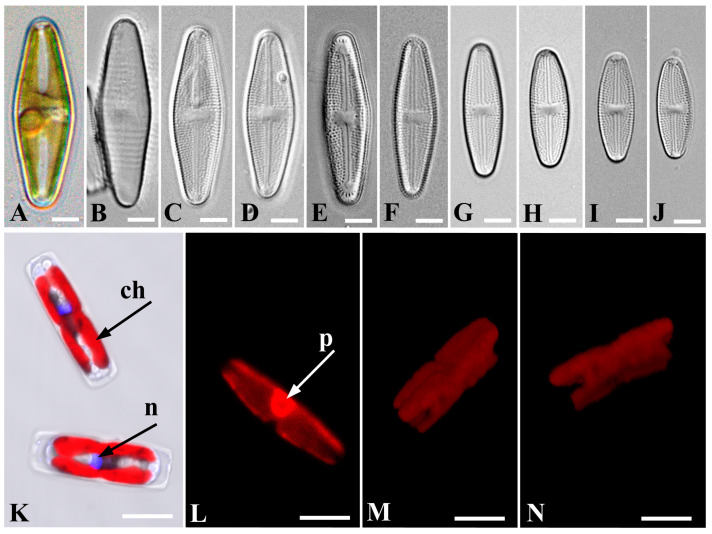
*Luticola tenera* sp. nov. LM (**A**–**J**): (**A**)—live vegetative cell; (**B**)—perizonium; (**C**–**J**)—valves of vegetative cells. CLSM (**K**–**N**): (**K**)—valve in girdle view with a double-H-shaped chloroplast (ch) and nucleus (n) (black arrows); (**L**)—chloroplast with a pyrenoid (p) (white arrow) in valve view; (**M**,**N**)—structure of a double-H-shaped chloroplast in 3D format. (**A**–**J**): Scale bar, 5 μm. (**K**–**N**): Scale bar, 10 μm.

**Figure 2:**
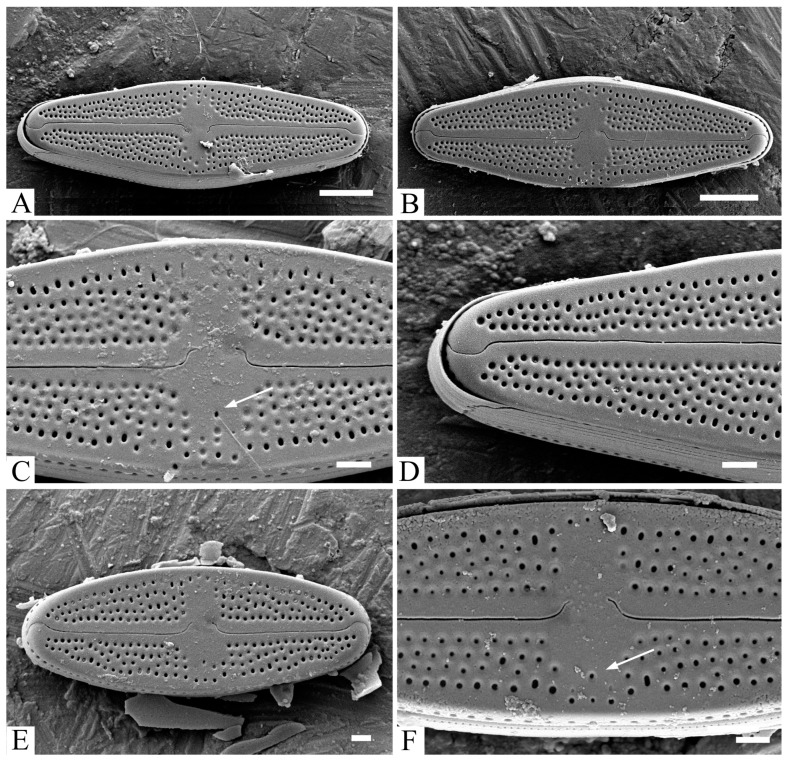
*Luticola tenera* sp. nov., SEM, external view. (**A**–**D**)—young vegetative cells; (**E**,**F**)—mature vegetative cells. (**A**,**B**,**E**)—general valve view showing composition of striae and typical raphe structure of a young vegetative cell; (**C**,**F**)—central area with “ghost areolae” and stigma (white arrow); (**D**)—apice of the valve, showing the distal raphe end. Scale bar: (**A**,**B**)—5 µm; (**C**–**F**)—1 µm.

**Figure 3:**
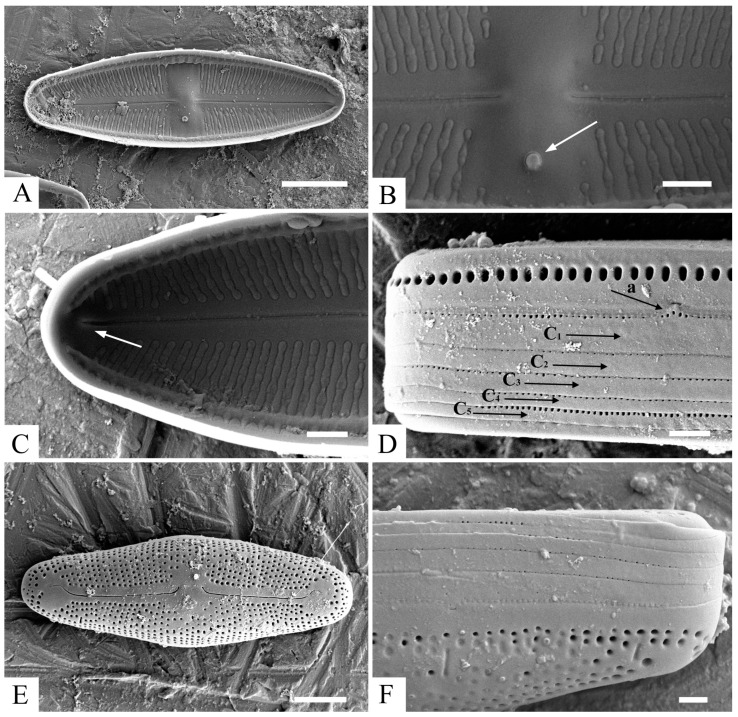
*Luticola tenera* sp. nov., SEM. (**A**)—general internal valve view showing composition of striae and typical raphe structure; (**B**)—central area with round stigma (white arrow) and internal valve view; (**C**)—distal raphe end with small helictoglossa (white arrow); (**D**)—structure of cingulum: raised mantle, exposing a valvocopula (black arrow “a”), C_1_—valvocopula, C_2_–C_5_—copulae (black arrows); (**E**,**F**)—structure of initial cell. Scale bar: (**A**,**E**)—5 µm; (**B**–**D**,**F**)—1 µm.

*Luticola tenera* Bagmet, Abdullin, A. Nikulin, V. Nikulin, and Gontcharov, sp. nov. Figure 1A–N, Figure 2A–F and Figure 3A–F.

Holotype: Exsiccatum number VLA-CA-1377, a dried biomass of the unialgal population, was deposited in the Herbarium, Federal Scientific Center of East Asian Terrestrial Biodiversity, Vladivostok, Russia. Gene sequence: DNA sequences obtained from clonal strain of *Luticola tenera* were deposited in the GenBank under accession no. OR326853.

Type locality: State Nature Reserve “Bastak”, Jewish Autonomous Region, Russia (48°25′59.5″ N 134°13′18.1″ E), soil.

Etymology: The species epithet “tenera” is based on the morphology of the valves.

Distribution: So far, it is only known from the State Nature Reserve “Bastak” (waterlogged soil).

Comment: It differs from other *Luticola* species based on the following set of morphological characters: the shape and width of the valve and hook-shaped external proximal raphe ends deflected to the side opposite to stigma. Also, genetically distinct due to differences in the chloroplast *rbc*L gene.

Description.

LM (Figure 1A–J). Live cells are solitary. Valves from rhombic-lanceolate and lanceolate with widely rounded, non-retracted ends (Figure 1A–F) to elliptic-lanceolate with slightly retracted, broadly rounded ends (Figure 1G–J). Valve dimensions (*n* = 40): length 15.7–35.0 μm; width 6.6–11.0 μm. Striae radiate, clearly visible in LM, 17–23 in 10 μm. Central area rectangular, wide. Axial area linear, narrow. Raphe straight. Proximal raphe endings poorly visible. The perizonium, formed by transverse bands (Figure 1B), is clearly visible in the LM.

CLSM (Figure 1K–N). Frustules rectangular in girdle view (Figure 1K). Chloroplast complex, H-shaped in girdle view, lying with its center along the secondary side of the girdle (Figure 1K,M–N). Pyrenoid single, oviform, in the center of the chloroplast near the girdle (Figure 1L). The interphase nucleus is displaced towards the primary frustule side, positioned eccentrically (Figure 1K).

SEM, external view (Figure 2A–F). Axial area narrow, expanded to the valve center (Figure 2A,B,E). Central area covered with “ghostly areolae”, making it slightly asymmetrical, rounded, or rectangular and bordered at each margin by 3–5 isolated rounded areolae (Figure 2C,F). Raphe straight (Figure 2A,B,E). Distal raphe ends hooked, deflecting towards same side as the proximal ends and then hooking towards opposite side (Figure 2D). Proximal raphe ends weakly asymmetrical, hooked, or just deflected opposite to the stigma (Figure 2C,F).

SEM, internal view (Figure 3A–C). Distal raphe ends branches terminating with small helictoglossae (Figure 3C). Proximal raphe ends straight, terminating at the edge of the stauros. Isolated pore single, round, close to the valve margin (Figure 3B). Raphe branches straight (Figure 3A). Areolae occluded by hymenes, forming a continuous strip across the valve (Figure 3B,C). Striae weakly radiate, 17–23 in 10 µm, composed of 2–6 rounded areolae. Marginal channel located on valve face/mantle junction, occluded with hymenes (Figure 3A).

SEM, cingulum (Figure 3D). Valve mantle with a single row of elongated areolae. A mature cingulum consists of five copulae, each bearing a number of rounded areolae of the same morphological structure, 55–60 in 10 µm. Valvocopula (C_1_) is the widest copula, and the second (C_2_) and the third (C_3_) copulae and the fourth (C_4_) and the fifth (C_5_) copulae are approximately equal in width.

No teratological forms were observed.

### 3.2. Phylogenetic Analyses

**Figure 4:**
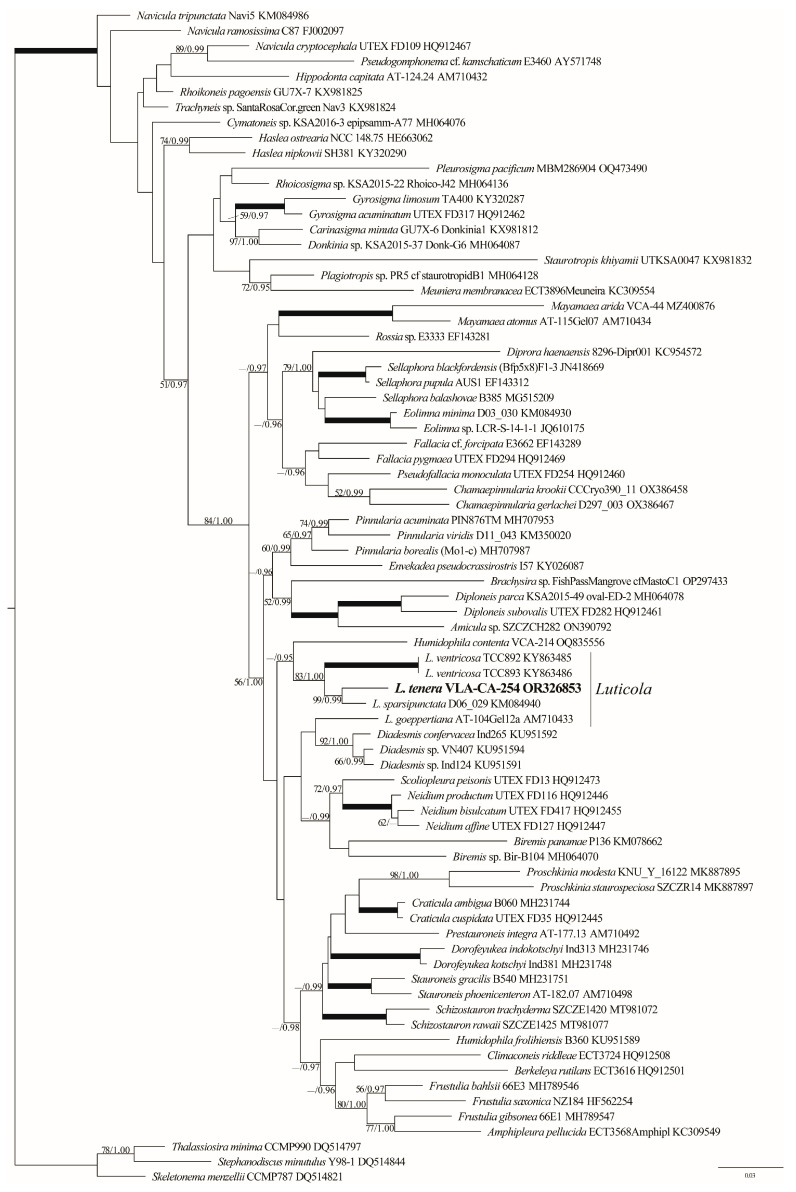
ML phylogenetic tree of the Naviculales (GTR+I+G model) showing the position of the new strain based on partial *rbc*L gene sequence data (77 sequences, 1434 aligned positions). Supports [(BP) > 50% and (PP) > 0.95: ML/BI] are provided above/below the branches. New strain and branches with 100% BP and 1.00 PP are shown in boldface.

The phylogenetic analyses of 77 *rbc*L sequences representing all available families of the Naviculales resulted in the tree presented in Figure 4. The topologies of the ML and BI trees based on the *rbc*L dataset were similar, except for some differences in the clade supports.

Genus *Luticola* was resolved paraphyletic and split into two lineages. *L. ventricosa, L. sparsipunctata,* and *L. tenera* formed a clade (83/1.00), while *L. goeppertiana* was topologically found to be a sister of *Diadesmis* spp. *L. tenera* was close to *L. sparsipunctata* with high support (99/0.99; Figure 4).

### 3.3. Sexual Reproduction

Homothallic reproduction was observed in the monoclonal culture of *L. tenera* with two types of gamete mating: normal and reduced.

**Figure 5:**
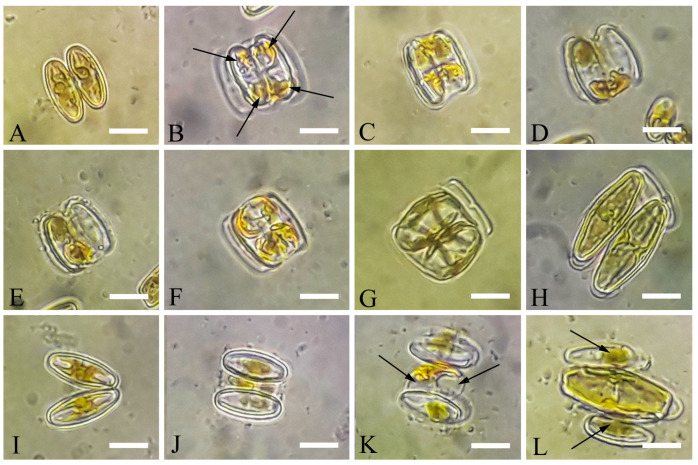
Sexual reproduction of *Luticola tenera* sp. nov., LM: (**A**–**H**)—Cis-anisogamy, normal type, (**I**–**L**)—isogamy, reduced type. (**A**,**I**)—pairing cells; (**B**)—gametogenesis (gametes are indicated by black arrows); (**C**–**E**)—syngamy, (**F**)—extended zygote with two chloroplasts; (**G**)—auxospores expanding perpendicular to the apical gametangial axes; (**H**)—initial cells; (**J**)—syngamy outside of the valves; (**K**)—auxospores with two nuclei (black arrows); (**L**)—auxospores expanding parallel to the apical axes of gametangia (black arrows indicate residual bodies). Scale bar: 10 µm.

Normal type: Two cells align side-by-side (girdle-girdle), forming a gametangial pair (Figure 5A). The cells become stationary after adhesion, and meiosis begins. It is followed by cytokinesis, which results in the formation of two gametangia. The protoplast of each gametangium transapically divides, forming two morphologically identical gametes.

The gametes are rounded and diverge toward the ends of the cell (Figure 5B). Two mobile gametes are formed in one gametangium, and two immobile gametes form in another. Motile gametes move to the gametangium containing immobile gametes (Figure 5C–E). As a result of syngamy, two zygotes are formed (Figure 5F). The zygotes bipolarly expand and elongate perpendicular to the valves of the parent cells (Figure 5G), turning into auxospores (Figure 5H). This type of sexual reproduction could be classified as cis-anisogamy. It designated as IA2a, according to Geitler [48], or 2 + (2)/ap + ap/1 + 1/ra, according to Davidovich and Davidovich [49].

Reduced type: After the formation of gametangia as described above (Figure 5I), gelatinous material is released by both cells, and a structure similar to the “copulation channel” is formed as a result of this material merging (Figure 5J,K). In each gametangium, two morphologically identical gametes are formed: one is active and another is passive. Then, two active gametes move towards each other through the copulation canal and merge. Non-motile gametes remain in the gametangia and die after a while. As a result of syngamy, one zygote is formed, which bipolarly extends parallel to the valves of the parent cells and turns into a diploid auxospore (Figure 5K,L). After some time, the auxospore aborts one nucleus, becoming haploid. This type of sexual reproduction is called isogamy. It designated as IC, according to Geitler [48], or 1. + 1./ap + ap/1 + 1/pa, according to Davidovich and Davidovich [49].

The auxospore perisonium is formed by transverse elements (Figure 1B). The initial cell is formed inside of the fully grown auxospore that morphologically differs from vegetative cells (Figure 3E). The shape of the initial cells differed from that of the first post-initial cells. The initial cells were rounded; there was no clear distinction between the external valve and the mantle. The number of areolae forming striae in the central area was greater (from 5 to 10) than in the post-initial cells (4–6). It is likely that some of these areolae subsequently form large oval poroids on the mantle, since they are absent in the initial cells (Figure 3F). The central area is small and rounded but bordered by many areolae (4–6 rows), some of which are likely become “ghostly” in post-initial cells. A fully formed initial cell emerges by breaking the perizonium and begins active vegetative division.

## 4. Discussion

### 4.1. Morphology

The diatom genus *Luticola* comprises taxa sharing the following morphological properties: distinctly punctate striae; a conspicuous isolated pore in the central area, commonly referred to as a ‘stigma’; and internal, longitudinal, and marginal canals on each side of the valve [8].

European *Luticola* species were revised by Levkov et al. [8]. These authors distributed 200 taxa between 17 artificial groups (A–Q) based on a combination of eight morphological features (length and width, shape, apices, stria density in 10 μm, central area, axial area, distal raphe ends, and proximal raphe ends). This grouping disregarded the geographic distribution of the species and their ecology. The morphological features of the new species fit the characteristics of group D; therefore, we compared its morphology to that of the group members, as well as to some other recently described species (Table S1). Of these, eight species are the most similar to *Luticola tenera*: *L. terrestris* Kochman-Kędziora, M. Rybak and Peszek; *L. asymmetrica* M. Rybak, Kochman-Kędzi and Peszek; *L. rojkoviensis* Hindáková and T. Noga; *L. darwinii* Witkowski, Bak, Kociolek, Lange-Bertalot and Seddon; *L. fuhrmannii* Metzeltin and Levkov; *L. hustedtii* Levkov, Metzeltin and A. Pavlov; and *L. intermedia* (Hustedt) Levkov, Metzeltin and A. Pavlov, *L. acidoclinata* Lange-Bertalot. All of these taxa are small celled (maximal length of ≤ 36 µm) and characterized by rhomboid-lanceolate valve [8].

Despite having a similar appearance, the new species is distinct in a broader valve (up to 11 µm). In all valves examined, ghostly areolae were observed. The same feature is also typical of *L. terrestris* and *L. asymmetrica*. Valves with the maximal length in *L. terrestris, L. asymmetrica*, and *L. darwinii* were rhomboid in shape, while in the new species, they were rhomboid-lanceolate in nature. Moreover, valves in *L. terrestris* are almost two times narrower, and its striae are formed by fewer areolae (from two to four). Cells with maximal length in *L. rojkoviensis* and *L. beyensii* have rhomboid-lanceolate valves like *L. tenera*; however, cells with the smallest lengths differ from those in our taxon. They are elliptic-lanceolate in *L. tenera* but rhomboid-lanceolate in *L. rojkoviensis* and *L. beyensii*. *L. fuhrmannii* and *L. acidoclinata* differ from *L. tenera* in the central area shape (from quadrangular to bow tie), and there are fewer areolae in striae (from three to five in *L. fuhrmannii* and from 3 to 4 in *L. acidoclinata*). *L. intermedia* and *L. tenera* differ in terms of the shape of the proximal raphe ends (deflected, expanded into a central pore vs. hook-shaped). Thus, the combination of morphological features (valve shape and width, shape of the proximal raphe ends, etc.) differentiates our taxon from earlier-described *Luticola* species.

During its life cycle, *L. tenera* varies not only in terms of the valve shape (from rhomboid-lanceolate to lanceolate and elliptic-lanceolate; Figure 1C–J), but also the number of areolae forming striae (Figure 2C,F; Table S1). Young vegetative cells with maximal cell lengths have four to six areolae near the central area, while cells with minimal length only have three to four areolae. A similar trend was described in representatives of the genera *Nupela* Vyverman and Compère*, Diadesmis* Kütz.,* Chamaepinnularia* Lange-Bertalot and Krammer*, Eunotia* Ehr.,* Decussata* (R.M. Patrick) Lange-Bertalot,* Luticola* D.G. Mann*, Microcostatus* J.R. Johansen and J.C. Sray, and *Nitzschia* Hassall inhabiting soils [50]. It is likely that valve perforation depends from its size and surface area to decrease water evaporation [50]. For ghostly areolae present in valves during the whole life cycle, however, their number decreases with the decreasing dimensions of the cells. Thus, a morphological feature like the number of areolae, forming the striae, is not constant in *Luticola* and cannot be used as a species-specific character.

### 4.2. Phylogeny

Up to date many diatom species were solely described based on morphological data (e.g., *Surirella caljoniana* Solak, Cocquyt and P.B. Hamilton [51]; *Planothidium africanum* Van de Vijver et al. [52]; *Orthoseira helvetica* Peszek et al. [53]). However, only a combination of morphological and molecular data reveals features, differentiating the new taxon, localizes it in the phylogenetic tree and fills databases with new sequences [54,55] that are still limited for diatoms.

Sequence data are only available for 5 out of 264 *Luticola* species, and only for 3 of them (*L. goeppertiana, L. sparsipunctata*, and *L. ventricosa*) was the *rbc*L gene sequenced. This finding may suggest difficulties in obtaining the cultures of the species.

Rather limited molecular data resolved the genus *Luticola* as paraphyletic because *L. goeppertiana* showed affinity with the *Diadesmis* Kütz. of the same family Diadesmidaceae (Figure 4). This topology contradicts the results presented by Kulikovskiy et al. [56] because in their tree, the genus clade was well supported. Our phylogeny revealed *L. tenera* to be a sister to *L. sparsipunctata*; however, these taxa are not morphologically close to each other. It is likely that a very limited sampling is unable to access the genus concept and relationships between its species with confidence.

### 4.3. Sexual Reproduction

The presence of two types of the sexual reproduction (normal and reduced) in *L. tenera* is intriguing. Typical anizogamous sexual reproduction, resulting in the formation of two auxospores (IA2a), was earlier described in *L. dismutica* [31] and *L. poulickovae* [29]. It is likely that this type is common in the genus. The IC reduced type was documented in *Luticola* for the first time here, but it was already observed in heterothallic *Schizostauron* sp. [57]. In *Schizostauron*, like in *L. tenera*, each mating cell produced two gametes, of which only two fused and the remaining two died. Such mating behavior could be explained by several reasons: inbreeding, too small cell sizes, the long distance between the mating cells, and not enough energy for successful syngamy gamete motility. Unlike *Schizostauron*, *L. tenera* is homothallic and forms a gelatinous “copulation channel” for active gamete movement. A similar type of the gametes merging was described in *Cocconeis pellucida* Grun. [58], but in this species, only one gamete per gametangium was formed that corresponded to isogamy (Geitler’s type IIB).

The presence of normal and reduced reproduction in one population, as we described for *L. tenera*, was reported for *Achnanthes longipes* Ag. [59]. A complex mating system with differentiation on monoecious, dioecious, and bisexual clones was revealed during the study of sexual reproduction in this species. Normal sexual reproduction prevailed (91% of the pairs studied), while the IC reduced type was only observed in 9% of the mating pairs. The appearance of the reduced type in *A. longipes* could be due to the smaller than normal type size of the mating cells or, more likely, inbreeding in a dense culture. It was shown that secondary inbreeding rapidly reduced viability, followed by the reduction in the gamete number from two to one in each gametangium and only one auxospore formation [60,61]. In *L. tenera*, parental cells were of similar length (ca. 16–17 µm) during both normal and reduced reproduction; therefore this, parameter did not affect a number of the mating gametes. IC type was observed after finishing the normal type reproduction (5–7 days after initiation), when most parental cells were already mating and cell density, including not yet mating parental cells and already dividing initial cells in the Petri dishes, was high. This result likely led to a high probability of inbreeding and the degradation of one gamete in the gametangium. Thus, *L. tenera* has an additional type of sexual reproduction, namely isogamy, for the maintenance of viability and species existence in unfavorable conditions.

## 5. Conclusions

We described a new member of the genus *Luticola*, *L. tenera* derived from the Russian Far East using the polyphasic approach. We characterized morphology variation and sexual reproduction in the species during its life cycle and showed that its phenotypic features differentiate *L. tenera* from those of other described members of the genus.

## Data Availability

The data presented in this study are available on request from the corresponding author. In addition, the data that support the findings of this study are openly available in GenBank.

## Supplementary Materials

The following supporting information can be downloaded via this link: https://www.mdpi.com/article/10.3390/life13091937/s1, Table S1: Comparative analysis of morphological and morphometric parameters in *Luticola tenera* and morphologically similar species.
