# Supplementary material for: Luticola tenera sp. nov. (Diadesmidaceae, Naviculales)—A New Diatom from the Soil of the State Nature Reserve “Bastak” (Jewish Autonomous Region, Russia)
Source: Life (Basel). 2023 Sep 20;13(9):1937. doi: 10.3390/life13091937 (PMC10533167; doi:10.3390/life13091937)
Supplement: Supplementary file 1 [file life-13-01937-s001.zip › life-2613276-supplementary.pdf]

Table S1. Comparative analysis of morphological and morphometric parameters in *Luticola tenera* and morphologically similar species.

	L. terrestris	***L. asymmetrica***	***L. rojkoviensis***	***L. darvinii***	***L.beyensii***	***L. fuhrmannii***	***L. intermedia***	***L. acidoclinata***	***L. tenera* sp.nov.**
Length, µm	8.0–28.5	12.5–36.0	11.7–32.0	18.0–28.0	17.0–30.0	18.0–34.0	16.0–29.9	10.0–30.0	15.7–35.0
Width, µm	4.4–6.1	6.0–8.0	6.0–9.0	6.0–8.0	6.5–9.5	7.5–8.5	5.8–8.5	5.0–8.0	6.6–11.0
Shape	lanceolate to rhombic-lanceolate	lanceolate, elliptic–lanceolate to rhombic lanceolate	rhombic–lanceolate to rhombic–elliptic	elliptical-lanceolate to lanceolate	rhombic-lanceolate	lanceolate to rombic-elliptic	lanceolate to rhombic-lanceolate	rhombic–lanceolate to lanceolate	rhombic–lanceolate to elliptic–lanceolate
Apices	usually rounded, in larger valves, slightly subcapitate, slightly protracted	usually rounded, in larger valves, slightly subcapitate, slightly protracted	slightly protracted to broadly rounded	with very weakly protracted, broadly rounded ends	larger individuals with subcapitate ends	rounded, not protracted	rounded	slightly protracted	slightly protracted to broadly rounded
Stria density (in 10 µm)	radiate throughout, 20–23	radiate throughout, 17–20	radiate throughout, 18–25	radiate throughout, 20–22	slightly radiate in the middle to radiate at the ends, 22–24	radiate, 22–24	radiate, 21–24	radiate, 21–24	radiate throughout, 17-23
Central area	bow-tie-shaped, often asymmetrical, bordered by shortened striae	bow-tie-shaped	weakly asymmetrical, wedge–shaped	nd	with a large fascia, rarely reaching the valve margins	rectangular to bow-tie-shaped	nd	wedge–shaped	weakly asymmetrical, rectangular
Axial area	linear, slightly widening towards both the central area and the apices	linear, slightly widening towards both the central area and the apices	narrow, linear, slightly expanded near central area and very slightly lowered	narrow, expanded at valve centre to wide, nearly-rectangular fascia	linear, relatively broad	narrow, linear, weakly expanded in the central area	narrow, linear	narrow, linear	linear, slightly widening towards both the central

Distal raphe ends	first curved to the same side as the proximal raphe fissures, then slightly bent to the isolated pore-bearing side	hooked towards opposite side, terminating shortly before valve edge	in the shape of a question mark up to a bayonet–shape in larger cells	long, question mark shaped, extending onto valve mantle	curved twice, first in the same direction as the central endings (primary side) and then returning towards secondary side	hooked	hooked and continuing onto the valve mantle	hooked	in the shape of a question mark, extending onto valve mantle
Proximal raphe ends	deflected away from the isolated pore-bearing side with small drop-like endings	long, unilaterally deflected to the side opposite to stigma and expanded into small pores	slightly deflected to opposite side to stigma	double curved long fissures	nd	deflected, expanded	deflected, expanded into a central pore	hooked	hook-shaped, deflected to side opposite to stigma
Ghost areolae	often present	present	nd	nd	nd	nd	nd	nd	often present
Number areolae bordered central area	nd	3–5	3–5	4–5	3–5	3–4	nd	3–5	3–5
Number striae composed areolae	2–4	2–5	2–7	nd	2–4	3–5	4–5	3–4	3–6
Habitat	terrestrial moss collected from rock	terrestrial moss collected from rock	rock environment is formed by the Mráznica Formation	floating moss	dry soils and mosses	nd	nd	nd	terrestrial
Locality	Prince Alfreds's Pass, Knysna, Western Cape, South Africa	Prince Alfreds's Pass, Knysna, Western Cape, South Africa	grassland of the hill in the village of Rojkov, Veľká Fatra, Slovakia	Ecuador. Galápagos islands: isabela (albemarle) island, diablas	Island of Saint-Paul, Amsterda m	Amany Ost-Usambaragebirg e Korogwrtal, Tanga, Tanzania	nd	Lake Tanganyica,M Pulungu, Ile Niamcolo, stone.	Soil, State Nature Reserve «Bastak», Jewish Autonomous Region,

				wetlands					Russia
Source	Rybak et. al., 2021	Rybak et. al., 2021	Hindáková & Noga, 2021	Bąk et al, 2019	Van de Vijver et al., 2002	Levkov et al., 2013	Levkov et al., 2013	Levkov et al., 2013	this study

Notes: nd – no data.
